# An Account of a Singular Fact, in the Practice of Inoculation of the Small Pox

**Published:** 1786

**Authors:** John Dawson

**Affiliations:** Surgeon at Sedbergh, in Yorkshire


XV. *An Account of a fingular Fact, in the Practice of Inoculation of the Small Pox. By Mr.* John Dawfon, *Surgeon at Sedbergh, in York-fhire.* Vide *Medical Tranfactions, publifhed by the College of Phyficians in London, Volume the Third,* 8vo. London, 1785.

THIS is the fact alluded to by Dr. Wright, in the preceding article.—Mr. Dawfon having inoculated two children in one family, obferved, on the third day, a flight inflammation around the places of incifion. On the fifth day the inflammation was confidera-bly increafed, and on the eighth it extended nearly to the breadth of half a crown.

With

With matter taken from the arms of these children, at this period, he inoculated nineteen other persons, and every one of these had a fever and eruption of pustules at a proper time; but the two children from whom the matter had been taken, did not sicken, as was expected; and on the eleventh day, the inflammation upon their arms was considerably abated; and two or three days after this there remained nothing but a dry scab.

In conformity to an opinion hitherto generally adopted, our author now ventured to assure the parents that their children were secure from future infection of the small pox; but they having insisted on their being again inoculated, a second incision was made in the arms of each. A fresh inflammation succeeded around the places of incision, and went on in the same manner it had done before, till about the ninth or tenth day, when the patients sickened, and had a smart fever during three days, after which appeared a considerable number of variolous pustules.

